# Design and implementation of ParkinsonAKTIV: an interventional study to evaluate the effectiveness of a novel online platform to guide quickcard-based treatment decisions

**DOI:** 10.1186/s42466-023-00249-5

**Published:** 2023-06-01

**Authors:** Katharina Achtert, Tessa Huchtemann, Maria Altendorf, Linda Kerkemeyer, Malte Haring, Carina Lummer, Lena Frenz, Theresa Becking, Jonas Friedmann, Philip Mildner, Katharina Schwarze, Lars Steinhaus, Volker Amelung, Tobias Warnecke

**Affiliations:** 1 Institute for Applied Health Services Research (inav GmbH), Schiffbauerdamm 12, 10117 Berlin, Germany; 2 Department of Neurology, University of Münster, Albert-Schweitzer-Campus 1, 48149 Münster, Germany; 3 ECONUM Unternehmensberatung GmbH, Martin-Luther-Str. 69, 71636 Ludwigsburg, Germany; 4 Nuromedia GmbH, Schaafenstr. 25, 50676 Cologne, Germany; 5 AOK Nordwest, Kopenhagener Straße 1, 44269 Dortmund, Germany; 6 Institute for Social Medicine, Epidemiology and Health Systems Research, Medical University Hannover, Karl-Wiechert-Allee 3, 30625 Hannover, Germany; 7 Department of Neurology and Neurorehabilitation, Klinikum Osnabrück – Academic Teaching Hospital of the University of Münster, Am Finkenhügel 1, 49076 Osnabrück, Germany

**Keywords:** Parkinson’s disease, Efficacy, Economic evaluation, Process evaluation, Social network analysis, Neurodegenerative disease, Innovative care approach, Nonpharmacological treatment, Study protocol

## Abstract

**Introduction:**

Patients with Parkinson’s Disease (PD) require an all-encompassing and individualized care including pharmacological as well as non-pharmacological treatment approaches, such as physical therapy, occupational therapy and speech and swallowing therapy. ParkinsonAKTIV is an innovative, multidisciplinary, and comprehensive approach to guide this non-pharmacological PD treatment in northwestern Germany. Its online communication platform called JamesAKTIV has been developed to enhance and standardize PD healthcare professionals’ communication. The implementation of ParkinsonAKTIV and JamesAKTIV is accompanied through a detailed process evaluation and to gather evidence on the impact on patient-related outcomes, such as health-related quality of life and healthcare costs for people with PD through an effectiveness evaluation.

**Methods:**

The study design contains two parts: (1) first, a quantitative effectiveness evaluation is conducted utilizing a prospective quasi-experimental approach with a control group which examines PD patient’s health-related quality of life and physician-assessed PD patient’s health status (Unified Parkinson Disease Rating Scale). Moreover, a health economic evaluation of the ParkinsonAKTIV intervention is conducted using patient-reported outcomes and cost data as well as routine data from a statutory health insurance. (2) Second, a mixed-methods process evaluation among healthcare professionals, which examines the feasibility and potential barriers and facilitators of ParkinsonAKTIV for routine care, is performed. Quantitative results from a social network analysis and a survey among healthcare professionals will be triangulated with data from qualitative stakeholder interviews and focus group discussions.

**Perspective:**

Findings are expected to provide evidence of an increase in quality of life of patients with PD, less severe PD symptoms, and a better ability to participate in activities of daily living. ParkinsonAKTIV has the potential of increasing PD patients’ quality of care through sufficient and more tailored prescription of non-pharmacological therapies. It is anticipated that ParkinsonAKTIV will improve communication among health professionals. Results from the ParkinsonAKTIV study will provide first practice-oriented evidence and a roadmap for implementation of an online tool for a comprehensive, multidisciplinary care PD network for patients and their caregivers in routine care in Germany.

*Trial registration* ClinicalTrials.gov: registration number NCT05251298 (retrospectively registered: https://clinicaltrials.gov/ct2/show/record/NCT05251298).

## Introduction

Parkinson’s disease (PD) is the second most common neurodegenerative disorder. Its course is generally slowly progressive and results in a variety of motor and non-motor symptoms [18]. In 2019, worldwide, approximately 8.5 million people have been affected by PD [16, 18]. Patients with PD (PwP) require a comprehensive and individualized management including medications, deep brain stimulation, and non-pharmacological treatments, such as physical therapy, occupational therapy as well as speech and swallowing therapy [4]. International network approaches have been shown to improve quality of life, reduce hospital admissions, and minimize the general burden of disease for PwP and their caregivers [11]. Notably, a study based on German routine data from a statutory health insurance revealed that there is a nationwide lack in non-pharmacological therapies for PwP [6]. Due to the fragmented German healthcare system with its inadequate collaboration and communication between healthcare professionals, an unmet need for comprehensive PD treatment approaches exists. Hence, an innovative treatment approach is needed to address and bridge these gaps in communication and collaboration through including a wide range of healthcare professionals, intimately involved in a community-care setting.

The *ParkinsonAKTIV* study, described in this paper, implements and evaluates such an innovative, multidisciplinary and comprehensive treatment approach for PwP in a region in northwestern Germany. As an essential part of the ParkinsonAKTIV study, an online communication platform to enhance PD healthcare professionals’ communication, called *JamesAKTIV*, is built and evaluated. The ParkinsonAKTIV study is set out to reach two aims: (1) first, to understand the implementation process and care process of ParkinsonAKTIV (and JamesAKTIV) through a detailed *process evaluation*. (2) Second, to gather evidence on the impact on patient-related outcomes, such as health-related quality of life and healthcare costs for PwP, through an *effectiveness evaluation*.

## Methods

### Study design

The present study follows a hybrid type 1 study design [19], focusing on implementation process factors and outcomes in the context of an effectiveness trial in a prospective quasi-experimental study with control group. A mixed-methods process evaluation among healthcare professionals examines the feasibility and potential barriers and facilitators of ParkinsonAKTIV for routine care, as well as the communication within the regional multidisciplinary Parkinson’s Network Muensterland (PNM+) consisting of medical and non-medical professionals involved in the treatment of patients with PD. For this purpose, quantitative results from a social network analysis and a survey among healthcare professionals will be triangulated with data from qualitative stakeholder interviews and focus group discussions.

Further, the quantitative effectiveness evaluation with two study arms (i.e., intervention arm and observational control arm) examines PD patient’s health-related quality of life and physician-assessed PD patient’s health status (e.g., with the Unified Parkinson Disease Rating Scale (UPDRS)). Moreover, a health economic evaluation of the ParkinsonAKTIV intervention is conducted using patient-reported outcomes and routine data from a German statutory health insurance.

The study observation period is set to 12 months, and data for the effectiveness evaluation and the associated health economic evaluation will be collected at five timepoints, as shown in Table 1.

**Table 1:** Trial flow-chart according to SPIRIT guidelines with schedule of enrollment, intervention, and assessment

Timepoint	Study period					
	Preceding study	Enrolment/allocation	Post-allocation			Close-out
	− *t*_1 (12 months prior)_	0	*t*_1_	*t*_2_	*t*_3_	*t*_4 (12 months)_
*Enrolment*						
Eligibility screen		X				
Informed consent		X				
Allocation		X				
*Interventions*						
ParkinsonAKTIV						
Control Group (treatment as usual)						
*Assessments*						
Demographic aspects		X				
PDQ-39		X		X^a^		X
FIMA		X		X^a^		X
Schwab & England—activities of daily living scale		X^a^	X^a^	X^a^	X^a^	X^a^
UPDRS		X^a^	X^a^	X^a^	X^a^	X^a^
Hoehn & Yahr scale		X^a^	X^a^	X^a^	X^a^	X^a^
Routine data	X	X	X	X	X	X

PDQ-39 Assessment of patient-reported health-related quality of life with the Parkinson’s Disease Questionnaire. *FIMA* assessment of patient-reported healthcare costs. *UPDRS* assessment of patient’s PD-related status through neurologists

^a^Only assessed in the intervention group

This study is registered at ClinicalTrials.gov (registration number NCT05251298). Ethical approval has been given by the ethics committee of the Medical Association Westphalia-Lippe and the University of Muenster (approval number: 2021-356-f-S).

### The intervention: development of the online platform JamesAKTIV

An essential part of the ParkinsonAKTIV study is the development and use of the online platform called JamesAKTIV. Various professions, such as neurologists, physiotherapists, occupational therapists, speech and language pathologists, PD nurses, representatives of a German statutory health insurance, and IT specialists were involved in the development of JamesAKTIV. One major feature of JamesAKTIV is the digital quickcard for each patient which aims to establish guideline-based non-pharmacological therapy recommendations and to enhance structured communication between health professionals involved in care for patients with PD. The development process of quickcards is described in detail elsewhere [8].

JamesAKTIV contains the following features:

- Quickcards that can be filled out and shared between neurologists and therapists.
- A comments function for quick exchange of relevant information.
- A download section to access assessment forms.
- Individual patient accounts for therapy information and administration.
- A video section explaining the ParkinsonAKTIV project in detail.
- Educational videos for specific therapeutic interventions.

### Use of JamesAKTIV

**Fig. 1:**
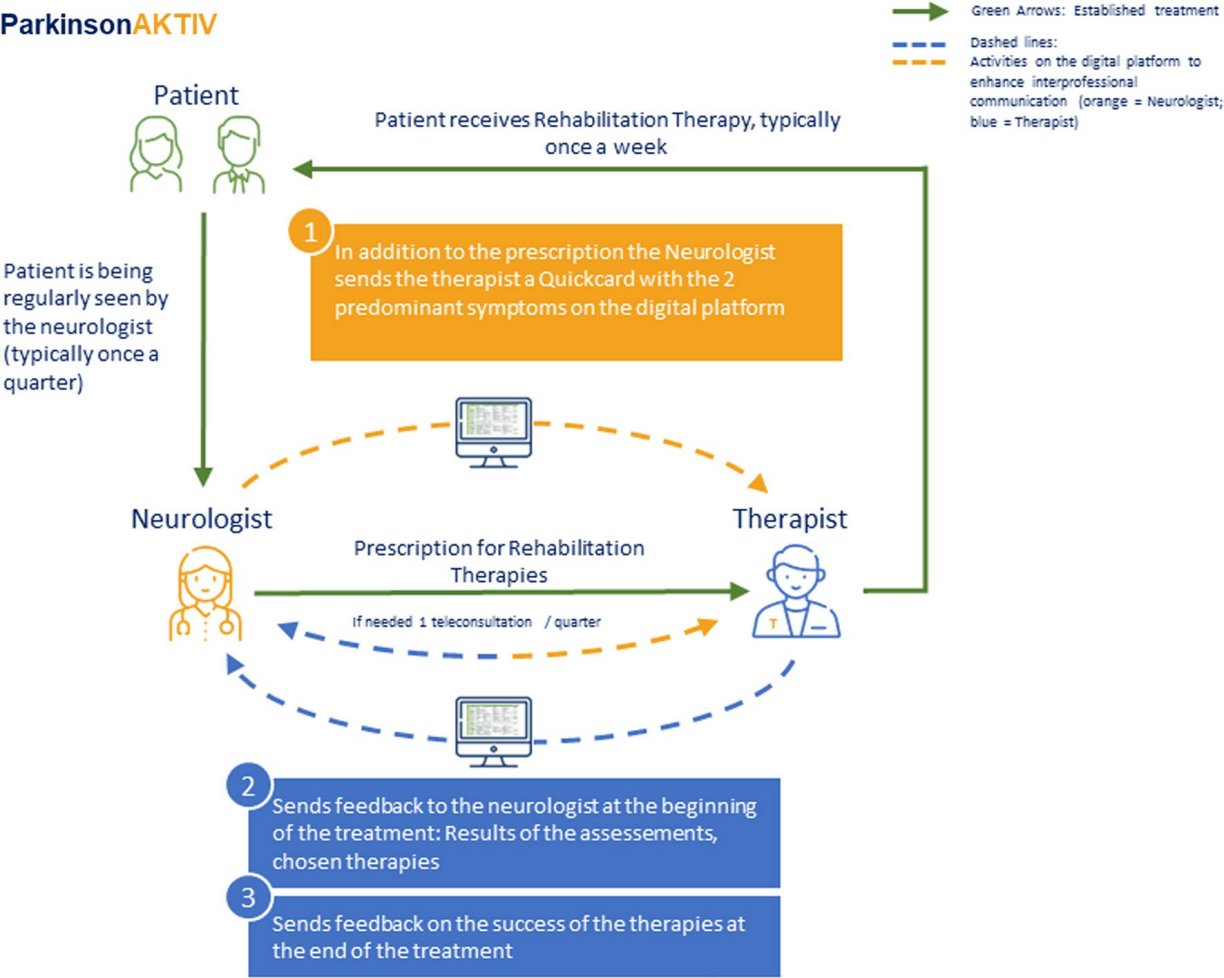
Illustration of JamesAKTIV

As illustrated in Fig. 1, treating neurologists log into JamesAKTIV and create a case for the PD patient under treatment. When the treating neurologist subscribes a non-pharmacological treatment, such as physiotherapy to their patient, he or she documents this treatment on JamesAKTIV as specific as possible. After an assessment by the therapist, a final decision is established between the neurologist and the therapist. Once the patient receives personalized treatment by a therapist, the kind of therapy (e.g., backpack training for camptocormia) is again documented in the quickcard by the physiotherapist and the progress, duration and the kind of therapy can be monitored by the treating neurologist.

### Study setting and training of staff

The intervention takes place in a specified area in the northwest of Germany (Muensterland and Osnabrueck). In this area, two multidisciplinary voluntary care networks of PD healthcare professionals exist, namely the PNM+ and the Parkinson’s Network Osnabrueck+(PNO+). Participating therapists and neurologists were recruited via these regional networks as well as from outside the networks in the region. All professionals participating in ParkinsonAKTIV are required to complete a 2-h education course on the use of JamesAKTIV. Moreover, all professionals working in ParkinsonAKTIV have had training on the use of quickcards and on diagnostics as well as treatment options in PD.

### Process evaluation

*Recruitment* Participants for the process evaluation are healthcare professionals located in the region of Muensterland, Tecklenburger Land and Osnabrueck, Germany (n = 30 neurologists and n = 150 therapists). All healthcare professionals are given access to JamesAKTIV and are invited for the quantitative process evaluation (i.e., social network analysis survey and the team effectiveness survey) via email for participation in the online survey. For the qualitative process evaluation (i.e., interviews and focus groups), a purposive sample will be drawn from all participating ParkinsonAKTIV healthcare professionals.

*Social network and team effectiveness analysis* A quantitative social network and team effectiveness analysis is performed using an online survey to analyze patterns of relationships among stakeholders within the system and to map the connection and provide insights into communication and collaboration between project partners. Another part of the online survey includes 25 questions on perceived team effectiveness, which cover aspects about psychosocial team characteristics and norms, dynamics and processes, such as communication, decision-making and potential conflicts within the team, and team outcomes [17].

*Interviews and focus groups* For semi-structured interviews, approximately, 10 – 15 healthcare professionals from ParkinsonAKTIV are approached and asked to participate in telephone interviews. The interviews aim to shed light on factors such as the acceptance of ParkinsonAKTIV (i.e., JamesAKTIV) for routine care implementation, willingness to work with JamesAKTIV, as well as its barriers and facilitators. In addition, focus groups with participating healthcare professionals are conducted to investigate interdisciplinary communication between different healthcare professionals and to gain insights into the quality of network interactions.

*Data analysis* Quantitative data gained from the social network analysis are analyzed descriptively and visualized in form of nodes and ties in a graphical network that depicts the relationships between involved healthcare professionals [11]. The relationships are grouped into five dimensions of analysis: Interconnectedness: how and where interaction occur, Centrality: number of interactions with other stakeholders, Density: ratio of actual and theoretical interactions between stakeholders, Distance: whether there are indirect or direct interactions between stakeholders, and Reachability: how many steps one stakeholder takes to reach another stakeholder.

Quantitative data from the team effectiveness evaluation is analyzed descriptively by computing mean scores of all items within one concept. Then an overall sum score from all concepts will be calculated reflecting the perceived level of team effectiveness [17].

The qualitative data collected in semi-structured interviews and focus groups, consisting of field notes and transcripts of audiotapes will be analyzed according to the principles of qualitative content analysis [10]. Subsequently, the quantitative and qualitative data from the process evaluation will be triangulated.

### Effectiveness evaluation

*Recruitment of intervention group* Recruitment of PwP takes place during face-to-face consultations by their treating neurologists where further information about ParkinsonAKTIV is provided. Until the caseload is reached, all new patients that fulfil the inclusion criteria, will be enrolled into the intervention arm. Additional information about the procedure for participation is available on the PNM+ website as well as flyers.

*Recruitment of control group* To compare the treatment of PwP in ParkinsonAKTIV with standard care, members of a nationwide support group for PD, the Deutsche Parkinson Vereinigung e. V. (dPV), are approached for study participation through their member’s journal. The dPV member pool will be approached twice for participation in the survey, once for the baseline survey and once at the end of the study.

Patients eligible for the intervention arm must fulfill the following inclusion and exclusion criteria:

*Inclusion criteria*

1. Primary PD according to the international classification (ICD 10 G20.x)
2. Age of ≥ 30 years
3. Existing medical treatment
4. PD care must be fully provided in the region Muensterland, Tecklenburger Land and the district and city of Osnabrueck

*Exclusion criteria*

1. Secondary PD
2. Insufficient knowledge of the German language
3. Intellectually and linguistically unable to answer the questionnaires

*Data collection* For the effectiveness evaluation and the health economic evaluation, quantitative data will be collected via paper–pencil surveys among PD patients (i.e., primary outcome: health-related quality of life and secondary outcomes: patient-reported healthcare costs, see Table 1). In addition, neurologists assess disease specific measures during their face-to-face consultations (i.e., secondary outcomes: disease severity and PD symptom severity, see Table 1).

*Sample size calculation* The sample size calculation is based on the primary outcome, quality of life. According to a-priori power calculation, based on an independent t-test and an effect size of d = 0.3 [3, 11]. With an alpha of 0.05 and a two-tailed comparison of means, a sample size of 176 PwP for each group would be sufficient to achieve a power of 0.80. In total, a case number of 352 PwP is needed due to the 1:1 matching. Considering a loss to follow-up in the intervention arm (estimated at 5%) [2], a total of 185 PwP needs to be initially included. PwP in the control groups will be surveyed via dPV. One study has been identified that recruited PwP via dPV with a resulting response rate of approximately 11% [12]. Considering a similar response rate of 11% for the ParkinsonAKTIV study and dPV’s current approximately 19,000 members across Germany, the inclusion of 185 PwP in the control arm appears to be reachable.

*Primary outcome* The primary outcome is health-related quality of life, assessed using the Parkinson’s Disease Questionnaire (PDQ-39, [1]). The PDQ-39 consists of 39 questions, subdivided into eight dimensions including items on symptoms, opportunities to participate, mobility and interferences in activities of daily living. Each question can be answered with following categories: “never” (= 0 points), “rarely” (= 1 point),” sometimes” (= 2 points), “often” (= 3 points) and “always” (= 4 points). A higher score indicates a reduction in quality of life (PDQ-39, [1]).

*Secondary outcomes. * The severity of PD symptoms will be assessed using the UPDRS, which evaluates various aspects of PD [5]. This instrument includes four different categories containing Part I: Non-Motor Aspects of Experiences of Daily Living, Part II: Motor Aspects of Experiences of Daily Living, Part III: Motor Examination and Part IV: Motor Complications. The questionnaire will be conducted during an interview with the respective patient by the treating neurologist. The UPDRS total score ranges from 0 to 199 points. A higher score indicates a physical or cognitive impairment of PwP [5].

The symptom progression of PD will be assessed by the treating neurologist using the Hoehn and Yahr scale [7], which comprises eight different disease stages. A higher disease stage indicates a higher limitation due to the PD [9].

The severity of PD and activities of daily living will be assessed using the PD-specific Schwab and England Activities of Daily Living scale [14] by the treating neurologist. This scale rates the functional status of patients with PD on a scale from 0, indicating worst possible function, to 100, indicating no impairment.

Healthcare service utilization will be measured using the generic Questionnaire for Health-Related Resource Use in an Elderly Population (FIMA) [15]. It contains 28 questions that consider the patients’ utilization and healthcare costs of outpatient and inpatient treatments, medications, and other treatment procedures, such as physical therapy.

*Health economic evaluation* The health economic evaluation will be performed as a cost-effectiveness analysis considering total costs (direct and indirect costs) collected from patients via FIMA and quality of life as effect measures collected via PDQ-39. In addition, a cost analysis from a payer’s perspective will be performed using routine data from a statutory health insurance.

*Data analysis* Descriptive and associated analyses will be conducted to determine sample characteristics and provide summaries of outcome measures.

The primary and secondary outcomes will be evaluated according to the intention-to-treat principle. To test the robustness of the data, per-protocol analysis with complete cases only will be performed.

For the analysis of the primary outcome measure, PD-specific quality of life, all 39 items will be grouped into eight index scores, which then represent a percentage value between 0 and 100 (100 = many health problems). In a next step, linear models will be used to examine the difference in primary outcome between groups and will be reported as gain in quality of life within the 12-monthts observation period, with a 95% confidence interval. Sensitivity analyses will be conducted using multiple regression analysis, considering covariates such as age, gender, or duration of PD.

Secondary outcomes will be analyzed with dependent t-tests, to compare for potential differences in the intervention group within the course of the study (12 months).

Cost-effectiveness will be analyzed using an incremental cost-effectiveness ratio (ICER). The ICER measures the additional cost per unit of health gain and compares the differences between the intervention arm (specified as ‘i’ in the formula) and control arm (specified as ‘c’ in the formula) in terms of the mean total costs in the numerator and mean effects in the denominator:

$${\text{ICER}} = {{\left( {{\text{costs}}_{{\text{i}}} - {\text{costs}}_{{\text{c}}} } \right)} \mathord{\left/ {\vphantom {{\left( {{\text{costs}}_{{\text{i}}} - {\text{costs}}_{{\text{i}}} } \right)} {\left( {{\text{effects}}_{{\text{i}}} - {\text{effects}}_{{\text{i}}} } \right)}}} \right. \kern-0pt} {\left( {{\text{effects}}_{{\text{i}}} - {\text{effects}}_{{\text{c}}} } \right)}}$$

To address uncertainties in the cost-effectiveness analysis, cost-effectiveness planes will be plotted based on a non-parametric bootstrapping method with 5.000 bootstrap replications. Furthermore, a cost-effectiveness acceptability curve (CEAC) will be constructed and univariate sensitivity analyses will be performed.

## Perspective

To the best of our knowledge, this is the first study testing and evaluating the implementation of a multidisciplinary treatment coordination platform for PwP in Germany. The ParkinsonAKTIV study aims to test a multidisciplinary, digitalized approach called JamesAKTIV, including a wide range of healthcare professionals intimately involved in the community-based care for PwP. It is anticipated that ParkinsonAKTIV can overcome current barriers to communication and coordination within PwPs’ care paths with special regard to non-pharmacological therapies. The German PD care landscape is characterized by a fragmented system with different healthcare professionals caring for PwP, hence prescribing PD treatment to patients yet not communicating with each other about the respective patient. The multidisciplinary communication and coordination platform JamesAKTIV is designed to address communication barriers by using digitalized quickcards as means of communication about PwPs' treatment.

An outstanding strength of ParkinsonAKTIV is its study design. Combining quantitative and qualitative data collection through a process evaluation and an effectiveness evaluation, gaining deeper insights into patient’s quality of life and ParkinsonAKTIV’s cost-effectiveness. Specifically, during process evaluation, a social network analysis will be conducted which is, to our knowledge, a novelty in analyzing teamwork in multidisciplinary PD healthcare teams.

It is assumed that the results of this study will further strengthen ParkinsonAKTIV. First, the findings are expected to provide evidence of an increase in quality of life of PwP, less severe PD symptoms, and a better ability to participate in activities of daily living. Second, ParkinsonAKTIV has high potential of increasing PwPs’ quality of care through sufficient and more tailored prescription of non-pharmacological therapies. Third, improved communication among health professionals could lead to an increased patient safety.

Some potential limitations have to be mentioned, such as that no classical RCT study design has been used. This is due to the “natural” and “real-life” character as well as the complex network intervention of this study because outcomes from such studies need to be practically oriented and network effects need to be measured by innovative study designs. Therefore, a process evaluation has been integrated in the study design to shed light on effects of ParkinsonAKTIV for the regional PD care network. Moreover, for a smooth implementation of the JamesAKTIV platform, healthcare professionals have to adopt the quickcard-based workflow, which often seems to be a hurdle as new routines have to be learnt [13].

Results from the ParkinsonAKTIV study will provide first evidence and a roadmap for implementation of a comprehensive, multidisciplinary care network for PwP and their caregivers in routine care in Germany.

## Data Availability

The data will be deposited on a protected server of the Institute for Applied Health Services Research (inav GmbH). Access is strongly regulated even for study personnel. Owing to the difficulty of de-identification (routine care, qualitative data, etc.), individual participant data will not be shared publicly. Upon reasonable request that includes a methodologically sound proposal for the usage of data that is also approved by the responsible review committee data may be shared.

## Abbreviations

CEAC: Cost-effectiveness acceptability curve
dPV: Deutsche Parkinson Vereinigung e. V.
FIMA: Generic Questionnaire for Health-Related Resource Use in an Elderly Population
ICD-10: International Classification of Disease, Tenth Revision
ICER: Incremental cost-effectiveness ratio
PD: Parkinson’s Disease
PDQ-39: Parkinson’s Disease Questionnaire
PNM+: Parkinson’s Network Münsterland
PNO+: Parkinson’s Network Osnabrueck
PwP: Patients with Parkinson’s Disease
UPDRS: Unified Parkinson’s Disease Rating Scale

## References

[1] Berger K, Broll S, Winkelmann J, Heberlein I, Müller T, Ries V (1999). Untersuchung zur Reliabilität der deutschen Version des PDQ-39: Ein krankheitsspezifischer Fragebogen zur Erfassung der Lebensqualität von Parkinson-Patienten. Aktuelle Neurologie.

[2] Blanchet K, James P (2012). How to do (or not to do)… a social network analysis in health systems research. Health Policy and Planning.

[3] Curran GM, Bauer M, Mittman B, Pyne JM, Stetler C (2012). Effectiveness-implementation hybrid designs: Combining elements of clinical effectiveness and implementation research to enhance public health impact. Medical Care.

[4] Deuschl, G., Oertel, W., & Reichmann, H. (2016). Deutsche Gesellschaft für Neurologie S3-Leitlinie Parkinson-Syndrom, idiopathisch [23.07.2020]. Retrieved from https://register.awmf.org/de/leitlinien/detail/030-010

[5] Goetz CG, Tilley BC, Shaftman SR, Stebbins GT, Fahn S, Martinez-Martin P, Poewe W, Sampaio C, Stern MB, Dodel R, Dubois B (2008). Movement Disorder Society-sponsored revision of the Unified Parkinson’s Disease Rating Scale (MDS-UPDRS): Scale presentation and clinimetric testing results. Movement Disorders: Official Journal of the Movement Disorder Society.

[6] Heinzel S, Berg D, Binder S, Ebersbach G, Hickstein L, Herbst H, Lorrain M, Wellach I, Maetzler W, Petersen G, Schmedt N (2018). Do we need to rethink the epidemiology and healthcare utilization of Parkinson’s disease in Germany?. Frontiers in neurology.

[7] Hoehn MM, Yahr MD (1998). Parkinsonism: Onset, progression, and mortality. Neurology.

[8] Kerkemeyer L, Achtert K, Claus I, Happe S, Overbeck J, Kleen N, Palesch A, Schmuck C, Krouß S, Perick J, Depenbrock L, Warnecke T (2020). Quickcard-based approach to guiding specific nonpharmacological treatments in a German Parkinson’s network. Journal of Clinical Medicine.

[9] Martinez-Martin P, Skorvanek M, Rojo-Abuin JM, Gregova Z, Stebbins GT, Goetz CG, QUALPD Study Group (2018). Validation study of the hoehn and yahr scale included in the MDS-UPDRS. Movement Disorders.

[10] Mayring P, Fenzl T (2019). Qualitative inhaltsanalyse.

[11] Rajan R, Brennan L, Bloem BR, Dahodwala N, Gardner J, Goldman JG, Grimes DA, Iansek R, Kovács N, McGinley J, Parashos SA, McGinley J (2020). Integrated care in Parkinson’s disease: A systematic review and meta-analysis. Movement Disorders.

[12] Ritter VC, Bonsaksen T (2019). Improvement in quality of life following a multidisciplinary rehabilitation program for patients with Parkinson’s disease. Journal of Multidisciplinary Healthcare.

[13] Rouidi M, Elouadi A, Hamdoune A (2022). Acceptance and use of telemedicine technology by health professionals: Development of a conceptual model. Digital Health.

[14] Schwab, R. S. (1969). *Projection technique for evaluating surgery in Parkinson’s disease.* Paper presented at the Third symposium on Parkinson’s disease.

[15] Seidl H, Bowles D, Bock J-O, Brettschneider C, Greiner W, Koenig H-H, Holle R (2015). FIMA–Fragebogen zur Erhebung von Gesundheitsleistungen im Alter: Entwicklung und Pilotstudie. Das Gesundheitswesen.

[16] Tysnes O-B, Storstein A (2017). Epidemiology of Parkinson’s disease. Journal of Neural Transmission.

[17] Dijk-de Vries, A. N. V., Duimel-Peeters, I. G., Muris, J. W., Wesseling, G. J., Beusmans, G. H., & Vrijhoef, H. J. (2016). Effectiveness of teamwork in an integrated care setting for patients with COPD: development and testing of a self-evaluation instrument for interprofessional teams. *International Journal of Integrated Care, 16*(1). DOI:10.5334/ijic.2454 PMC5015529 27616953

[18] WHO. (2022). Parkinson disease. Retrieved from https://www.who.int/news-room/fact-sheets/detail/parkinson-disease

[19] Ypinga JH, de Vries NM, Boonen LH, Koolman X, Munneke M, Zwinderman AH, Bloem BR (2018). Effectiveness and costs of specialised physiotherapy given via ParkinsonNet: A retrospective analysis of medical claims data. The Lancet Neurology.

